# Ketamine in treating opioid use disorder and opioid withdrawal: a scoping review

**DOI:** 10.3389/fpsyt.2025.1552084

**Published:** 2025-04-30

**Authors:** Mary R. Shen, Dylan E. Campbell, Anika Kopczynski, Samuel Maddams, Naomi Rosenblum, Kabir Nigam, Joji Suzuki

**Affiliations:** Department of Psychiatry, Brigham and Women’s Hospital, Boston, MA, United States

**Keywords:** ketamine, opioid use disorder, opioid withdrawal, NMDA antagonist, substance use

## Abstract

**Introduction:**

Opioid use disorder (OUD) continues to be a public health crisis in the United States, with mortality having doubled over the last twenty years, leading to significant economic cost, morbidity, and mortality. This has caused significant demand for novel therapeutics. Preliminary evidence demonstrates that ketamine may be helpful in treating OUD as well as serve as an adjunct during treatment of opioid withdrawal (OW).

**Methods:**

We conducted a scoping review of two databases (PubMed and PsycINFO) for English language, peer-reviewed manuscripts reporting on use of ketamine in treatment of OUD or OW in humans, excluding protocols and reviews. The study was conducted in accordance with PRISMA guidelines.

**Results:**

The search yielded 998 studies. After duplicates were removed, 715 studies underwent title and abstract screening for inclusion in the review. Of those, 21 were further considered under full text review. Three studies were excluded due to wrong study design and ten were excluded due to the wrong indication, specifically the use of ketamine for treatment of pain rather than substance use disorder or withdrawal. Eight studies were included in the review, regarding treatment for OUD (n=2) and OW (n=6). In OUD, ketamine administration was helpful in reducing opioid cravings and opioid use. In OW, ketamine attenuated precipitated withdrawal symptoms and was used in several case series/studies as an adjunct to buprenorphine treatment.

**Conclusion:**

In summary, this review presents a baseline of literature supporting the use of ketamine in OUD and OW. However, more research is needed before widespread use.

## Introduction

Despite significant attention and ongoing efforts, opioid use disorder (OUD) continues to be a public health crisis in the United States. The economic burden of OUD is estimated to be over $1 trillion annually, driven by reduced quality of life from OUD and the value of life lost from fatal opioid overdose (1). While prevalence of OUD has remained stable since the 1990s, rates of death have increased 10-fold in the last twenty years, due to higher potency formulations of opioids, specifically in illicitly-sourced fentanyl (2, 3). Given the morbidity and mortality associated with OUD, there continues to be significant demand for novel therapeutics.

Ketamine has been approved for medical treatment since the 1960s, but its use has been limited to short-term sedation and anesthesia (4). In recent years, there has been increased interest in expanding the use of ketamine and its various forms, with FDA approval garnered for the enantiomer, esketamine, in treatment-resistant depression in 2019 (5). Since then, there have been continued efforts to expand the use of ketamine in other disorders, ranging from bipolar depression, PTSD, obsessive compulsive disorder, epilepsy, and fibromyalgia, as well as substance use disorders (6). Specifically, there is interest in exploring the use of ketamine not only in treating opioid use disorder (OUD) but also as an adjunct in the treatment of opioid withdrawal (OW) (7).

Ketamine is primarily classified as a N-methyl-D-aspartate (NMDA) receptor antagonist, affecting the glutaminergic pathways in the brain. However, it also has pleiotropic effects on a multitude of receptors and channels. Ketamine blocks nicotinic acetylcholine ion channels and affects monoamine transporters by inhibiting serotonin, dopamine, and norepinephrine reuptake (8). Furthermore, it is possible that there are interactions of ketamine with the opioid system, though it is not well understood (9). Specifically, in OUD, chronic opioid use leads to NMDA receptor upregulation in the mesolimbic pathway “reward” center. It is thought that NMDA antagonism decreases the activity of these pathways, increasing neuroplasticity and recircuiting the cue-response pathways in OUD (8). Moreover, it is possible that opioid withdrawal is mediated in part by the NMDA receptor. Additionally, it possible that ketamine’s mechanistic activity may involve the opioid receptor, facilitating its role in opioid withdrawal pathology. However, the opioid receptor effects of ketamine remain incompletely characterized. Preliminary animal studies have demonstrated that ketamine administration can also alleviate the symptoms of opioid withdrawal (9, 10).

This review aims to discuss the use and efficacy of ketamine in the treatment of OUD and OW in order to establish a baseline of literature in this sphere, as well as identify gaps in the literature to target future research.

## Methods

### Search strategy

We developed a search algorithm that included the terms ‘ketamine,’ as well as terms indicating OUD and OW, (i.e., ‘X’ use disorder, ‘X’ withdrawal, ‘X’ abuse, ‘X’ addiction, ‘X’ dependence), where ‘X’ represented opioid, fentanyl, and heroin). PubMed and APA PsychINFO were searched from database inception to 09/05/2024. Identification of studies for inclusion followed the Preferred Reporting items for Systematic Reviews and Meta-Analyses (PRISMA) guidelines (10). Articles identified were extracted and imported into Covidence, a systematic review platform. Additional articles were found through manual review of reference lists in identified articles that met the inclusion criteria.

### Inclusion/exclusion criteria

Our inclusion criteria comprised of English language, peer-reviewed manuscripts regarding use of ketamine in the treatment of OUD or OW in humans. Studies were excluded if they did not include humans, studied the illicit use of ketamine, or if the study did not discuss the use of ketamine in the treatment primarily of OUD or OW (e.g., including the use of ketamine in the treatment of pain in opioid-tolerant patients). Given the limited number of papers in this study, group consensus among researchers was to include case reports and case series. Study protocols and reviews were excluded.

### Study selection

Two authors (DC, SM) independently screened titles and abstracts. Two additional authors independently screened full texts (AK, NR). Senior authors (MS, JS) resolved conflicts independently after title and abstract screening, as well as after full text review. The study was conducted in accordance with PRISMA guidelines.

### Data extraction/analysis

Three authors (AK, SM, DC) extracted data from identified articles into an electronic database. Data extracted included population demographics, study design, interventions utilized (dosing, route, adjuncts), primary and secondary outcomes, and relevant results. A meta-analysis was not possible given the heterogeneity in design and intervention of identified studies.

## Results

**Figure 1:**
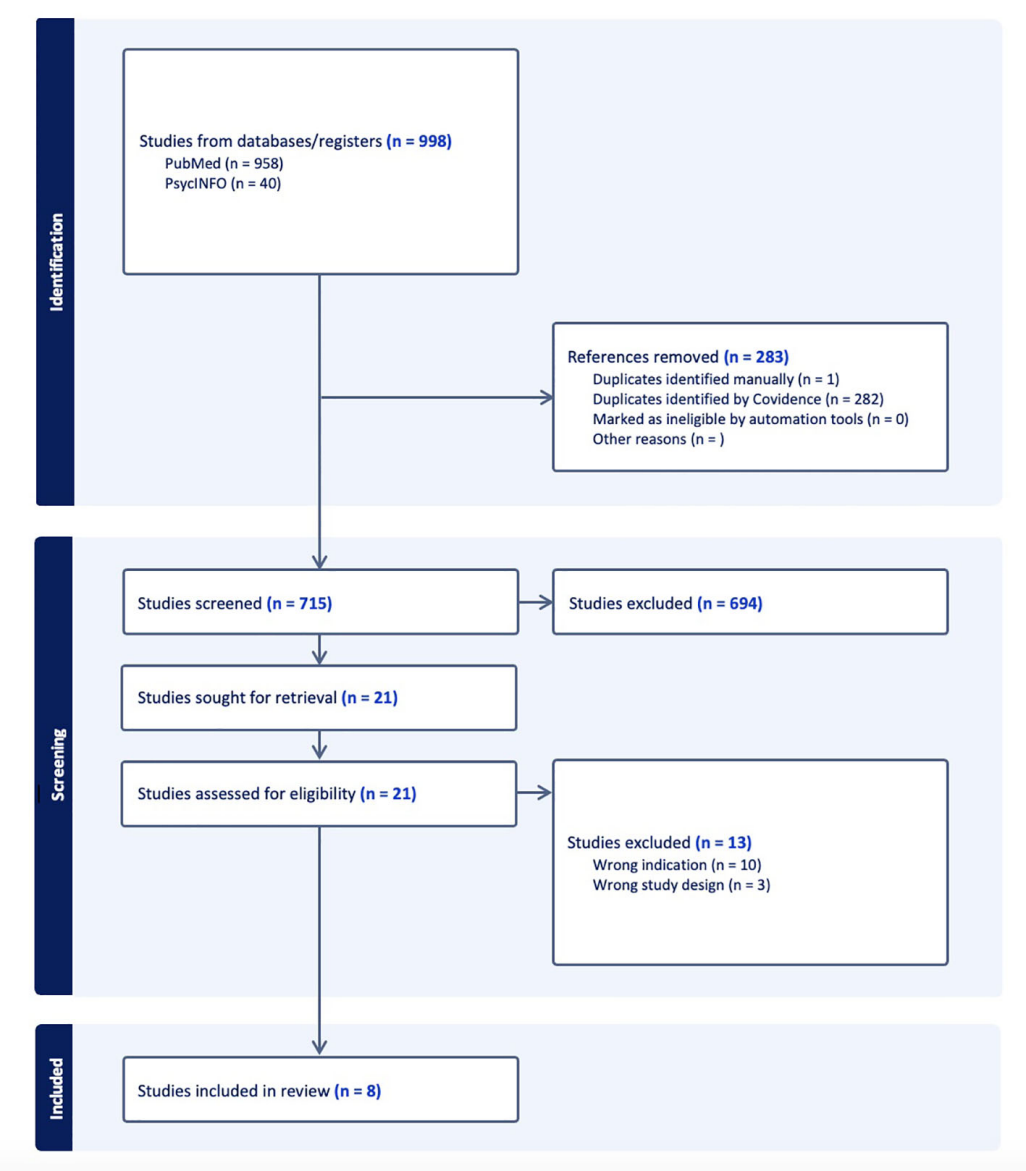
The PRISMA flow diagram for the scoping review detailing the database searches, the number of abstracts screened, the full texts retrieved, and the studies included.

The search yielded 998 studies. After duplicates were removed, 715 studies underwent title and abstract screening for inclusion in the review. Of those, 21 were further considered under full text review. Three studies were excluded due to wrong study design and ten were excluded due to the wrong indication, specifically the use of ketamine for treatment of pain rather than substance use disorder or withdrawal. Eight studies were included in the review (**Figure 1**), regarding treatment for OUD (n=2) and OW (n=6).

### Ketamine in the treatment of OUD

In examining the efficacy of ketamine in treating OUD, there were two randomized controlled trials (RCTs) identified (**Table 1**).

**Table 1:** Summary of studies on ketamine and opioid use disorder.

Study (Ref. #)	Population	Study Design	Intervention	Outcomes and Endpoints (primary and secondary)	Results
Krupitsky et al. (2002) (11)	• Adults with OUD (DSM-IV or ICD-10 criteria of opioid dependence for at least one year) aged 18-30 and abstinent from all “substances of abuse” for 2+ weeks • *n* = 70 • Age, mean 22.3 • 78.57% male) • Race not reported	• Randomized control trial (parallel group, double-blind, placebo-controlled) • Single-center: • St. Petersburg Regional Center of Addictions and Psychopharmacology, Russia	• High-dose group received 2.0 mg/kg ketamine intramuscularly Low-dose (active placebo) group received 0.20 mg/kg ketamine intramuscularly • All participants received a single ketamine session from a specially trained practitioner. • All participants received ten hours of psychotherapy before and five hours of psychotherapy after the ketamine session.	• Primary: • Abstinence until 24 months or relapse • Secondary: craving scale, depression scale, and anxiety scale	• High-dose group significantly more likely to be abstinent for up to two years post-treatment. Both groups showed significant craving reduction for up to 2 years (1.7 vs. 29.2, p<0.01). • Both groups experienced reduced anhedonia, anxiety, and depression with no significant differences between groups. • *Conclusion*: Ketamine-assisted psychotherapy of people with heroin addiction is more effective at prolonging abstinence when a high, psychedelic dose is administered compared to when a low, non-psychedelic dose is administered.
Krupitsky et al. (2007) (12)	• Adults inpatients at an addiction treatment hospital with OUD (DSM-IV or ICD-10 criteria of opioid dependence for at least one year) aged 18-35 and abstinent from all “substances of abuse” for 2+ weeks n = 59 • Age, mean 22.6 years 83.1% male Race not reported	• Randomized clinical trial • Single center: • St. Petersburg Regional Center of Addictions and Psychopharmacology, Russia	- Participants received either one or three ketamine-assisted psychotherapy sessions, all receiving 2.0 mg/kg intramuscularly each session. All received five hours of psychotherapy and five hours of psychotherapy after the first ketamine session. All participants received the initial ketamine session post-detox and pre-discharge, followed by two addiction counseling sessions at 1 and 2 months Those in the multiple-session arm received two more ketamine sessions, preceded by addiction counseling.	- Primary: Abstinence up to 12 months • Secondary: craving scale, Purpose-in-Life Test (PLT), a depression scale, and an anxiety scale	• At 1-year, 50.0% of participants in the three-session group were abstinent compared to 22.2% of the participants in the single-session group. • No significant differences in depression, state and trait anxiety, and craving scores between groups • *Conclusion:* The three-session program was more effective in promoting abstinence than the single-session program, despite also receiving similar standard addiction counseling.

Both RCTs, conducted by Krupitsky et al. in Russia, examined the use of ketamine in the setting of ketamine-assisted psychotherapy (11, 12). The first study, conducted in 2002, recruited 70 young adults with a history of opioid dependence as defined by the DSM-IV, who had been sober for at least two weeks (11). The researchers conducted a parallel group, double-blinded, placebo-controlled RCT examining abstinence rates, as well as symptoms of craving, depression, and anxiety. The intervention group received a “high-dose” or psychedelic dose of 2.0 mg/kg ketamine intramuscularly, while the “low-dose” or active placebo group received 0.2 mg/kg ketamine intramuscularly. According to the authors, a psychedelic dose was defined as a hallucinogenic dose, quantitatively assessed using the Hallucinogen Rating Scale. Patient scores at this level were consistent with those known to produce a comparable psychedelic experience to N, N-dimethyltryptamine (DMT) in this validated scale. In contrast, a low dose “elicit[ed] sub-psychedelic experiences and functions[ed] as ketamine-facilitated guided imagery.” (11) As this was ketamine-assisted psychotherapy, participants underwent ten hours of preparatory psychotherapy before the ketamine session and the ketamine session itself lasted between 1.5 to 2 hours. A five-hour integration therapy was offered thereafter to help participants process their experience. During the ketamine session, participants were made comfortable, offered eyeshades and music. The psychotherapist remained present throughout, offering support during the session. A single psychiatrist conducted all psychotherapy sessions. Participants were followed for 12 months with assessments that included urine toxicology. Results demonstrated that members of the “high-dose” group were significantly more likely to be abstinent at 1 and 2 months (p<0.01) and at all other months except 7 and 8 (p<0.05). Both groups showed reduced cravings, with the high-dose group experiencing greater reductions than controls immediately post-therapy (4.0 vs. 15.1, p<0.01), at 1 month (7.7 vs. 20.2, p<0.05), 3 months (5.4 vs. 28.3, p<0.01), and 1 year (1.7 vs. 29.2, p<0.01). While both groups experienced reduced anhedonia, anxiety, and depression, they were not significantly different between groups. These results demonstrate that a “high-dose,” psychedelic dose of ketamine was more effective at prolonging abstinence compared to “low-dose” or non-psychedelic dose of ketamine.

In 2007, Krupitsky et al. conducted a similar trial determining whether number of ketamine-assisted psychotherapy sessions impacted 1-year abstinence rates (12). They recruited 59 young adults and randomized patients into receiving either one or three ketamine sessions, with all participants receiving 2.0 mg/kg intramuscularly per session. After detoxification, participants received five hours of psychotherapy followed by the first ketamine session prior to discharge. After discharge, participants received five more hours of psychotherapy. Those randomized to receive a single session received two monthly addiction counseling, while those randomized to the three-session group received two more ketamine sessions each preceded by addiction counseling. At 12-months, rate of abstinence in the three-session group was greater compared to the single-session group (50% vs 22.2%, p<0.05), with no differences between depression, anxiety, and craving scores between groups. Of note, all symptom measures significantly decreased after the first session and continued to decline for both groups for those who remained abstinent and continued adherence to the protocol.

### Ketamine in the treatment of OW

There were six studies identified that examined the efficacy of ketamine in the treatment of OW (**Table 2**). Of these six studies, there was one prospective cohort study and one RCT. The remaining four studies identified were case series or single case studies. Two of the studies examined the effect of ketamine on withdrawal symptoms after receiving an opioid receptor antagonist (13, 14). Two case reports included patients receiving ketamine for treating buprenorphine precipitated opioid withdrawal (BPOW), and one case series reporting results using ketamine as a preventative measure while initiating patients on buprenorphine (15–17).

**Table 2:** Summary of studies on ketamine and opioid withdrawal.

Study (Ref. #)	Population	Study Design	Intervention	Outcomes and Endpoints (primary and secondary)	Results
Jovaisa et al. (2006) (14)	- Adult patients 18-35 with opioid dependence, otherwise healthy, as defined by DSM IV, duration of substance use more than one year n=58 Age, mean 22.7 (ketamine group), 23.4 (control group) - ages provided only by group - Race not reported	- Randomized, placebo-controlled, double-blind study - Single-center at Vilnius University Emergency Hospital, Lithuania.	- Intervention: Subanesthetic infusion of ketamine (0.5 mg/kg) administered after induction of anesthesia, 5 minutes before RAI - Control: Normal saline infusion - All patients underwent rapid opiate antagonist induction (RAI) under anesthesia. Patients underwent 48-hr postanesthesia phase treatment, then discharged to aftercare program (abstinence-based, naltrexone-supported outpatient counseling or residential rehabilitation programs)	-Primary: Withdrawal severity was measured according to Objective Opiate Withdrawal Scales (OOWS-A). -Secondary: - Mean arterial pressure (MAP) and heart rate (HR) - Cortisol levels - Medication requirements for symptom relief (i.e., clonazepam, carbamezepine) - Addiction Severity Index questionnaire at 4-months	• Opiate withdrawal scores were significantly lower in Ketamine group for 1st and 2nd hour post-RAI. Ketamine significantly reduced MAP, HR, and post-RAI morning cortisol levels • The ketamine group required significantly less clonazepam and carbamazepine in the 48-hr postanesthesia phase. • There were no significant differences in opioid abstinence rates between groups, and no significant outcomes from the 4-month follow-up were observed. *Conclusion*: Ketamine infusion suppressed the expression of precipitated opiate withdrawal and prevented significant rise in cardiovascular, respiratory, and neuroendocrine response especially during the first two hours of opiate antagonist induction. No significant differences across groups for relapse.
Freye et al. (2006) (13)	Adults enrolled in methadone maintenance programs or were regularly using heroin who had 5-10 unsuccessful inpatient detoxifications n=31 Age, mean = 31.7 74% male Race not reported.	Prospective cohort study Heinrich-Heine University, Dusseldorf, Germany	All patients pre-medicated with propofol, clonidine and somatostatin infusion, as well as naltrexone 50 mg administered 2x one hour apart. IV ketamine was administered at 1.5 mg/kg	Primary: Intensity of central excitatory effects (EEG-power spectra in the β, α, Θ, and Δ-band) after ketamine administration	Administration of S(+)-ketamine led to a reversal of acute abstinence-related changes in EEG power: compared to anesthesia with naltrexone, EEG power increased by 65% in the delta band and decreased by 723% in the beta band. *Conclusion:* While sympathetically induced hemodynamic alterations in anesthesia-assisted opioid detoxification can be attenuated by clonidine and sedation, central nervous sensory activation can be attenuated by the administration of S(+)- ketamine (1.5 mg/kg).
Grande et al. (2024) (17)	Adult patients transitioning to buprenorphine from fentanyl or methadone n=37 Age range 19-62 years (mean /median not provided). 54% female -Race not reported	Pilot case series Washington State, US	- Sublingual ketamine (16 mg) per administration, taken 2-3 times daily as needed for withdrawal symptoms for a total of 8 doses. - All patients were initiated on buprenorphine or buprenorphine/ naloxone with doses ≥8 mg within 24 hours for successful initiation. Clonidine, gabapentin, and other supportive medications were prescribed as needed to manage withdrawal symptoms or anxiety. No further counseling provided.	Primary: Successful buprenorphine initiation, defined as tolerating ≥8 mg of buprenorphine within 24 hours without worsening withdrawal symptoms, as measured by COWS.	• In patients who received ketamine, 67% (16/24) successfully completed buprenorphine initiation. • 92% of patients who completed initiation (11/12) achieved 30-day retention. • No serious adverse effects were noted. Conclusion: Ketamine prescribed at a sub-dissociative dose allowed for successful initiation of buprenorphine with reduced/elimination of opioid withdrawal symptoms.
Christian et al. (2023) (16)	38-year-old male patient with severe OUD presenting with suicidal ideation and withdrawal 24 hours after last fentanyl use	Case report (Yale New Haven Hospital, Connecticut, USA)	Patient was initially treated with sublingual buprenorphine-naloxone (BNX) 16–4 mg, exacerbating withdrawal symptoms, raising his Clinical Opiate Withdrawal Scale (COWS) score to over 36, then received two (24 and 36h) ketamine infusions at 0.3 mg/kg over 15 minutes, followed by a third ketamine infusion 0.3 mg/kg over one hour, along with additional BNX 8–2 mg.	COWS score	• After the first ketamine infusion, the patient's COWS score improved from over 36 to 18, though some symptoms remained. • A second ketamine infusion at the same dosage, along with additional BNX, completely resolved the patient's POW symptoms within 8 hours, and he was stabilized for discharge. • No severe side effects were noted. Conclusion: Ketamine shows potential to mitigate precipitated opioid withdrawal (POW) symptoms through μ-opioid receptor potentiation, making it a viable adjunctive treatment in hospitalized settings.
Hailozian et al. (2022) (15)	32-year-old male patient with opioid use disorder who developed severe buprenorphine precipitated opioid withdrawal (BPOW) from a failed outpatient microdose treatment plan.	Case report - Highland Hospital, Oakland, CA	Patient was treated with ketamine (0.6 mg/kg intravenous over 1 hour) and high-dose buprenorphine (16 mg sublingual single dose) followed by administration of month-long dose of extended-release subcutaneous buprenorphine which was repeated monthly for three months.	Primary outcomes: COWS, treatment adherence, and abstinence from opioid use disroder.	• One-hour post-ketamine administration, COWS decreased from 18 to 2. • At 120 days, patient remained in treatment and reported continuous abstinence from fentanyl use. • No severe side effects were noted. Conclusion: Ketamine is effective for reducing symptoms of precipitated opioid withdrawal and may have a role in continued abstinence as an adjunctive treatment to buprenorphine.
Lalanne et al. (2016) (18)	- 36 yo woman with severe opioid use disorder in context of lumbo-sciatic pain, presenting with a regimen of oxycontin extended-release 120 mg daily, oxycodone 60 mg daily, and acetaminophen/codeine 300 mg/25 mg 6 times per day).	Case report - University Hospital of Strasbourg, Strasbourg, France	Patient received ketamine oral solution 1 mg/kg, then opioid regimen was downtitrated by 10% every 48 hours Patient also receivd consultation with psychiatrist, addictologist, and pain specialist	Primary outcomes: Withdrawal symptoms	• No report of withdrawal symptoms (COWS Score of 0 out of 11), pain, or cravings while her opioid treatment was being reduced over 2 weeks. • No side effects of ketamine other than “unusual weakness.” • Pain regimen reduced to 50 mg codeine three times a day for pain. Conclusion: Withdrawal from ketamine was achieved without withdrawal symptoms.

In the only RCT, Jovaisa et al. examined 58 patients with opioid dependence as defined as the DSM-IV undergoing rapid opioid antagonist induction under anesthesia with tracheal intubation (14). Patients in the intervention group received a subanesthetic infusion of ketamine (0.5 mg/kg), finding that ketamine infusion significantly suppressed symptoms of precipitated opiate withdrawal especially in the first two hours after induction. This was true specifically in terms of peak mean arterial pressure (96.6 ± 13.8 in the placebo group vs. 79.4 ± 10.8 in the ketamine group, p<0.001) and peak heart rate (95.5 ± 12.8 in the placebo group vs. 75.6 ± 13.0 in the ketamine group, p<0.001). Furthermore, patients in the ketamine group required significantly less supportive medications (i.e., carbamazepine, clonazepam) to maintain the same level of opiate withdrawal symptoms during the first 48 hours (p<0.001).

Freye et al. conducted a prospective cohort study examining changes in electroencephalogram excitatory activity in acute withdrawal (13). The authors recruited adults actively using heroin or enrolled in methadone maintenance programs who had several unsuccessful inpatient detoxifications in the past to examine electroencephalogram excitatory activity. The authors found that S-ketamine infusion attenuated the increase in EEG activity and amplitude height of sensory-evoked potentials (p<0.01) during rapid opioid detoxification.

Grande et al. conducted a pilot case series in Washington State over 14 months detailing an iterative process of using ketamine to successfully transition adult patients with OUD from fentanyl or methadone to buprenorphine using either low- or macro-dose inductions (17). Patients self-administered 4-8 doses of sublingual ketamine 16 mg (3-6% of an anesthetic dose) with daily monitoring of symptoms. Of the included 37 patients, 16 patients (43%) successfully completed buprenorphine initiation. However, of the last 12 patients who completed buprenorphine initiation, 11 (92%) achieved 30-day retention in treatment. Patients who used ketamine reported reduction of elimination of spontaneous opioid withdrawal symptoms. The authors report that the novel strategy here is recommending patients to proactively use ketamine before emergence of withdrawal to facilitate buprenorphine induction.

The remaining three studies were single case reports. The first was a case report of a 36-year-old woman in France with severe OUD in the context of chronic back pain who was treated with ketamine in an attempt to reduce her daily opioid consumption (18). Prior to ketamine treatment, she was on a regimen of oxycontin extended-release 120 mg daily, oxycodone 60 mg daily, and acetaminophen/codeine 300 mg/25 mg 6 times per day). She received one oral dose of ketamine solution (1 mg/kg), then her opioid regimen was down-titrated by 10% every 48 hours to a goal of 50 mg codeine three times daily over two weeks. Patient experienced no withdrawal symptoms as her regimen was down-titrated with minimal side effects.

The single case reports by Hailozian et al. and Christian et al. both examined the effect of ketamine in the treatment of BPOW (15, 16). Given this is the terminology used by authors, we have elected to remain consistent while acknowledging that the precipitation is due to unregulated fentanyl use in the setting of using buprenorphine as evidence-based treatment. In the first case, a 32-year-old male patient with OUD, who initially presented to the emergency department with suicidal ideation, developed severe BPOW after lack of response to outpatient low dose induction, with COWS score reaching 18. Of note, he had several attempts to transition to buprenorphine in the outpatient setting, with the goal of abstinence, prior to presenting to the emergency department. He was treated with one infusion of IV ketamine (0.6 mg/kg over 60 minutes) along with high-dose buprenorphine (16 mg) administered sublingually, after which, patient’s COWS score decreased to 2. The patient was given month-long dose of extended-release buprenorphine delivered subcutaneously prior to discharge. The extended-release buprenorphine was repeated monthly for 3 months. At 120 days, the patient was able to remain in treatment and remained abstinent from opioid use (15). In the second case, a 32-year-old male patient with history of severe OUD undergoing withdrawal (24 hours since last fentanyl use) and concomitant suicidal ideation was initially given buprenorphine-naloxone (BNX) 16-4 mg over two doses sublingually in the emergency department, which raised his COWS score to 36+ (15). He first received one ketamine infusions (first 0.3 mg/kg over 15 minutes, immediately followed by 0.3 mg/kg over 60 minutes) along with additional BNX. After the first ketamine infusion, the patient’s COWS score was sustained at 18, then after the second ketamine infusion, the patient’s COWS score decreased to 0 within 8 hours (16). Of note, all studies examining use of ketamine for OW used standard adjunctive treatments, including anti-anxiolytics, analgesics, anti-emetics, among other medications.

## Discussion

In the last decade, several reviews have been published exploring the therapeutic use of ketamine in substance use disorders, eating disorders, obsessive-compulsive disorder, depression, bipolar disorder, suicidal ideation, social anxiety and generalized anxiety disorder, post-traumatic stress disorder, and ketamine’s interactions with electroconvulsive therapy (20–22). Ketamine has also shown preliminary efficacy in other substance use disorders, including alcohol use disorder and stimulant use disorder (19, 20). The findings from these studies are especially promising for stimulant use disorder, as there is a modicum of pharmacological treatments. In fact, a systematic review of ketamine treatment for stimulant use disorders found that ketamine improved cravings, motivation, and decreased cocaine use rates (19).

This is the first review, to our knowledge, that specifically examines the efficacy of ketamine in treating OUD and OW. While the amount of research in this space is clearly limited, one strength of this review is the inclusion of two RCTs in examining the use of ketamine in treatment of opioid use disorder. Furthermore, for treatment of OW, this review adds several successful recent case reports of using ketamine as an adjunct to buprenorphine to prevent withdrawal symptoms and precipitated withdrawal (16–18).

In the use of ketamine to treat OUD, we identified two RCTs, with both studies conducted by Krupitsky et al. The author found that increasing dose and number of ketamine treatments administered improved outcomes (i.e., rates of abstinence in patients receiving three ketamine-assisted psychotherapy sessions were almost twice as high as patients receiving one ketamine-assisted psychotherapy session) (12). This finding is consistent with the literature in using ketamine for other alcohol use disorder. In 2009, Kolp et al. conducted a retrospective cohort study finding that patients who received two ketamine infusions had higher alcohol abstinence rates compared to patients who received just one infusion (21).

In these RCTs examining ketamine for OUD, it is difficult to disentangle the effects of psychotherapy from the effects of ketamine in these RCTs, as all patients received some amount of ketamine, whether it was a “psychedelic” dose or a “non-psychedelic” dose. There are no RCTs with ketamine and opioid use disorder that have compared ketamine-assisted psychotherapy to only ketamine, which is a conspicuous gap in the literature. However, in looking to the literature in using ketamine in alcohol use disorder, Grabski et al. found patients receiving concomitant therapy had more days abstinent and lower odds of relapse, but the confidence interval included the null (22). Furthermore, as above, the retrospective cohort study by Kolp et al. found that patients undergoing more intensive group psychotherapy sessions had higher abstinence rates (21). As it stands, the evidence is limited - the role of psychotherapy in ketamine administration for opioid use disorder is currently unclear and requires further exploration.

In the studies examining the role of ketamine in OW, the findings suggest benefit in its effect on diminishing withdrawal symptoms and CNS sensory activation. As such, it is important to discuss two studies that discuss the proactive use of ketamine in opioid withdrawal, but did not meet inclusion criteria, as neither are peer-reviewed manuscripts. Casey et al. reported in a letter to the editor the use oral ketamine (20 mg dosed every 6 hours) to treat concurrent pain and opioid withdrawal (23). In 113 patients, they found the reduction of total oral morphine equivalents by 35% throughout the course of the hospitalization. Heeney et al. conducted a retrospective case series of 10 patients examining the use of ketamine for buprenorphine-precipitated opioid withdrawal. Patients generally received ketamine 0.3 mg/kg IV infusion over 15 minutes, followed by 0.3-1 mg/kg infusion over 1 hour. All patients were able to discharge safely from the hospital and the majority (8/10) remained engaged in treatment through California Bridge services (24).

It must be noted that a substantial barrier to research with psychoactive compounds is identifying an appropriate placebo for ketamine (25). In examining the role of ketamine in OW, two of the studies administered ketamine under anesthesia, patients were effectively blinded, which strengthens the interpretation of the results of those studies (13, 14). The potential utility of ketamine as an adjunct to treating OW, especially during buprenorphine induction, is of particular importance given the challenges patients now face in safely initiating treatment. More research is urgently needed to better understand how ketamine can mitigate the risk of OW during buprenorphine inductions.

Limitations of this review include heterogeneous studies with small sample sizes, restricting the ability to conduct a meta-analysis. This was especially true for examining the use of ketamine in OW, as the majority of the studies cited were case series/reports. Furthermore, there was heterogeneity in ketamine dosing, routes of administration, and adjunctive psychotherapeutic treatments, which may have greatly affected the outcomes. Furthermore, the dosing varied by study, an important consideration given difference in ketamine bioavailability based on route of administration (i.e., IV, IM, sublingually, or orally). For the RCTs exploring OUD, ketamine was administered intramuscularly (IM) at either a high (e.g., 2.0 mg/kg) or low dose (e.g., 0.20 mg/kg), or as the same dose (2.0 mg/kg) across single versus multiple sessions. Observational studies utilized intravenous (IV) ketamine (n = 4), sublingual ketamine (n = 1), or oral ketamine (n = 1). The differences in bioavailability and metabolism across these routes are important to discuss. IV administration results in 100% bioavailability and rapid onset, IM has approximately 93% bioavailability with slightly slower absorption, sublingual administration bypasses first-pass metabolism but has variable bioavailability (~32%), and oral administration has the lowest bioavailability (~17%) due to extensive first-pass metabolism (26, 27). As such, creating a standardized protocol in the future may be challenging without increasing homogeneity in dosing and route of administration in future studies. It is also important to acknowledge that 694 studies were excluded out of 712 non-redundant results. Given the dearth of research in this area, we opted for a more inclusive search to ensure no relevant studies were missed. Finally, in terms of the demographics of patients recruited, none of the studies reported race. In four of the five larger studies, the majority of the sample were aged 22-31, with male patients comprising 74-83% of the studied population, limiting generalizability of results. This point is especially true for the RCTs for OUD, where both studies were conducted in Russia, in a predominantly young, male population. Further research is needed to evaluate ketamine treatment across a more representative range of backgrounds and sexes.

As with the trials of ketamine for alcohol use disorder, it is unknown if combining ketamine treatment with mindfulness-based relapse prevention would lead to different outcomes. Another limitation is the challenge of isolating the effects of ketamine, particularly given its nature as a network therapeutic. For instance, it is known that patients with depression are more likely to use opioids long-term and that comorbid depression may drive substance use (28, 29). In fact, it is possible that there is endogenous opioid system dysregulation in depression (30). Thus, in examining OUD, it is difficult to extricate the effects of depression and/or other comorbid mental health disorders as a mediator for post-treatment abstinence. Furthermore, it is unclear how long the effects of the treatment is sustained, as the longest follow-up period was only 24 months (11). Interestingly, in the Jovaisa et al. study, there was no significant difference in length of abstinence from opioids at 4 months (14), which differed from the results demonstrated by Krupitsky et al. (11, 12). More research would be needed to see if abstinence is sustained, and if so, how long the effects last.

Additionally, the cost-effectiveness of ketamine treatment for OUD/OW has not been explored, thus more work needs to be done to clarify the financial feasibility. This point would be especially true if used as an adjunct to intensive psychotherapy, similar to the two RCTs examining ketamine for treating OUD.

It is important to consider how ketamine compares to standard care for OUD. Medications for opioid use disorder (MOUD), like methadone and buprenorphine, have well-established efficacy, with studies showing a 20-60% abstinence from opioids, according to a review examining randomized controlled trials of opioid-abstinence rates when using MOUD (31). Psychosocial interventions, including cognitive-behavioral therapy (CBT) and contingency management, have demonstrated success rates of approximately 30-50% when used alone but are more effective when combined with MOUD with studies demonstrating abstinence rates as high as 85% in randomized trials (32, 33). In the present review, for the treatment of OUD, the two studies included used ketamine as a replacement for MOUD. As such, there is a clear gap in the literature in examining the utility of ketamine as an adjunctive treatment to MOUD. Furthermore, it is known that utilization of MOUD in eligible populations is as low as 17% (34). It is not currently known if patients who are unwilling or unable to accept MOUD would be more willing to engage in ketamine-assisted psychotherapy. In clinical scenarios with limited opportunities to interface for treatment due to setting (i.e., a temporary setting like residential rehabilitation programs) or patient population (i.e., clients who are undomiciled or migrant workers), it would be helpful to further explore ketamine as a potential solution. Long-term comparative efficacy of these different treatments remains unclear. While psychotherapy remains a mainstay of addiction treatment, ketamine’s rapid-acting effects may provide a window of increased neuroplasticity that could enhance psychotherapeutic outcomes. Further research is needed to determine optimal integration strategies and long-term efficacy relative to standard care.

Though ketamine may demonstrate several benefits, there are also risks. When taken in high doses, it can have an anesthetic effect that impairs respiration and cardiovascular function, potentially leading to fatal consequences if misused, especially in unmonitored settings or when combined with CNS depressants or opioids (35). Hallucinogen use disorder can develop from ketamine use and long-term use has been associated with cognitive decline, including impairment of memory, as well as persistent dissociative or delusional thinking (36). Furthermore, there may be increased risk in ketamine-induced cystitis, leading to bladder scarring. This process is thought to be secondary to inflammatory signaling pathways causing damage to the bladder epithelium due to ketamine metabolites (37). While ketamine-induced cystitis (KIC) is most prevalent in illicit ketamine use, there have been case studies involving cystitis as a side effect of ketamine use for anesthetic and depression-treatment purposes (38, 39). Hemodynamic instability must also be monitored closely with higher risks for those with pre-existing cardiovascular conditions (40), though as seen in the studies listed above, ketamine may attenuate hemodynamic variability in patients undergoing precipitated opioid withdrawal (14). It is also unclear whether mode of administration can affect opioid use or withdrawal, as oral, intranasal, intravenous, and intramuscular forms exist. While none of the participants in these studies developed a hallucinogen use disorder, more research is needed to evaluate the safety of this treatment, as well as target populations that may derive the most benefit from this medication. Furthermore, there may be gaps in access to ketamine, such that those who may benefit the most would face significant financial barriers to treatment with ketamine (41).

These studies demonstrate promising evidence for the use of ketamine in treatment of OUD and OW. However, more research is needed. While there are currently five clinical trials in this space with unpublished results according to ClinicalTrials.gov, more research is needed to determine the optimal dose, frequency, and adjuncts of treatment (i.e., psychotherapy or other substance use treatment programs), as well as the safety and tolerability of this medication. Furthermore, given the recent approval for esketamine as monotherapy for treatment-resistant depression (42), the role of ketamine as a singular treatment versus as an adjunct for psychotherapy or medications for OUD requires further exploration.

## Conclusions

In summary, this review presents a baseline of literature supporting the use of ketamine in OUD and OW. In OUD, ketamine was shown to be effective as an adjunct to psychotherapy, with increasing efficacy for patients receiving higher doses and greater number of sessions. In OW, ketamine appears to be a helpful treatment, along with other supportive medications, in treating withdrawal symptoms and facilitating buprenorphine initiation. However, we strongly urge that more rigorous research is needed before ketamine is recommended for widespread use as a therapeutic for OUD and OW.

## Data Availability

The original contributions presented in the study are included in the article/supplementary material, further inquiries can be directed to the corresponding author/s.

## References

[1] Florence C Luo F Rice K . The economic burden of opioid use disorder and fatal opioid overdose in the United States, 2017. Drug Alcohol Depend. (2021) 218:108350. doi: 10.1016/j.drugalcdep.2020.108350 33121867 PMC8091480

[2] Rudd RA Aleshire N Zibbell JE Matthew Gladden R . Increases in drug and opioid overdose deaths—United states, 2000–2014. Am J Transplant. (2016) 16:1323–7. doi: 10.1111/ajt.13776 26720857

[3] Kuczyńska K Grzonkowski P Kacprzak Ł Zawilska JB . Abuse of fentanyl: An emerging problem to face. Forensic Sci Int. (2018) 289:207–14. doi: 10.1016/j.forsciint.2018.05.042 29902699

[4] Chang . The history of ketamine use and its clinical indications . SpringerLink. Available online at: https://link.springer.com/chapter/10.1007/978-3-319-42925-0_1 (Accessed November 21, 2024).

[5] Yavi M Lee H Henter ID Park LT Zarate CA . Ketamine treatment for depression: a review. Discovery Ment Health. (2022) 2:9. doi: 10.1007/s44192-022-00012-3 PMC9010394 35509843

[6] Morgan CJA Curran HV . Drugs (ISCD) the ISC on. Ketamine use: review Addict. (2012) 107:27–38. doi: 10.1111/j.1360-0443.2011.03576.x 21777321

[7] Nowacka A Borczyk M . Ketamine applications beyond anesthesia – A literature review. Eur J Pharmacol. (2019) 860:172547–7. doi: 10.1016/j.ejphar.2019.172547 31348905

[8] Ivan Ezquerra-Romano I Lawn W Krupitsky E Morgan CJA . Ketamine for the treatment of addiction: Evidence and potential mechanisms. Neuropharmacology. (2018) 142:72–82. doi: 10.1016/j.neuropharm.2018.01.017 29339294

[9] Gomes I Gupta A Margolis EB Fricker LD Devi LA . Ketamine and major ketamine metabolites function as allosteric modulators of opioid receptors. Mol Pharmacol. (2024) 106:240–52. doi: 10.1124/molpharm.124.000947 PMC11493337 39187388

[10] Page MJ McKenzie JE Bossuyt PM Boutron I Hoffmann TC Mulrow CD . The PRISMA 2020 statement: An updated guideline for reporting systematic reviews. Int J Surg. (2021) 88:105906. doi: 10.1016/j.ijsu.2021.105906 33789826

[11] Krupitsky E Burakov A Romanova T Dunaevsky I Strassman R Grinenko A . Ketamine psychotherapy for heroin addiction: Immediate effects and two-year follow-up. J Subst Abuse Treat. (2002) 23:273–83. doi: 10.1016/S0740-5472(02)00275-1 12495789

[12] Krupitsky EM Burakov AM Dunaevsky IV Romanova TN Slavina TY Grinenko AY . Single versus repeated sessions of ketamine-assisted psychotherapy for people with heroin dependence. J Psychoactive Drugs. (2007) 39:13–9. doi: 10.1080/02791072.2007.10399860 17523581

[13] Freye E Latasch L Levy JV . S(+)-ketamine attenuates increase in electroencephalograph activity and amplitude height of sensory-evoked potentials during rapid opioid detoxification. Anesth Analgesia. (2006) 102:1439. doi: 10.1213/01.ane.0000202382.82847.64 16632823

[14] Jovaisa T Laurinėnas G Vosylius S Šipylaitė J Badaras R Ivaškevičius J . Effects of ketamine on precipitated opiate withdrawal. Medicina (Kaunas). (2006) 42:625–34. 16963828

[15] Hailozian C Luftig J Liang A Outhay M Ullal M Anderson ES . Synergistic effect of ketamine and buprenorphine observed in the treatment of buprenorphine precipitated opioid withdrawal in a patient with fentanyl use. J Addict Med. (2022) 16(4):483–7. doi: 10.1097/ADM.0000000000000929 34789683

[16] Christian NJ Butner JL Evarts MS Weimer MB . Precipitated opioid withdrawal treated with ketamine in a hospitalized patient: A case report. J Addict Med. (2023) 17:488. doi: 10.1097/ADM.0000000000001151 37579118

[17] Grande LA Hutch T Jack K Mironov W Iwuoha J Muy-Rivera M . Ketamine-assisted buprenorphine initiation: a pilot case series. Addict Sci Clin Pract. (2024) 19:60. doi: 10.1186/s13722-024-00494-2 39210398 PMC11363367

[18] Lalanne L Nicot C Lang J-P Bertschy G Salvat E . Experience of the use of Ketamine to manage opioid withdrawal in an addicted woman: a case report. BMC Psychiatry. (2016) 16:395. doi: 10.1186/s12888-016-1112-2 27832755 PMC5105239

[19] Jones JL Mateus CF Malcolm RJ Brady KT Back SE . Efficacy of ketamine in the treatment of substance use disorders: A systematic review. Front Psychiatry. (2018) 9:277. doi: 10.3389/fpsyt.2018.00277 30140240 PMC6094990

[20] Martinotti G Chiappini S Pettorruso M Mosca A Miuli A Di Carlo F . Therapeutic potentials of ketamine and esketamine in obsessive–compulsive disorder (OCD), substance use disorders (SUD) and eating disorders (ED): A review of the current literature. Brain Sci. (2021) 11:856. doi: 10.3390/brainsci11070856 34199023 PMC8301752

[21] Kolp E Friedman HL Young MS Krupitsky E . Ketamine enhanced psychotherapy: preliminary clinical observations on its effectiveness in treating alcoholism. Humanistic Psychol. (2006) 34:399–422. doi: 10.1207/s15473333thp3404_7

[22] Grabski M McAndrew A Lawn W Marsh B Raymen L Stevens T . Adjunctive ketamine with relapse prevention–based psychological therapy in the treatment of alcohol use disorder. AJP. (2022) 179:152–62. doi: 10.1176/appi.ajp.2021.21030277 35012326

[23] Casey ER Hetrick ML Zwiebel SJ . Potential application of ketamine in pain and withdrawal in patients with opioid use disorder. J Acad Consult Liaison Psychiatry. (2024) 65:409–10. doi: 10.1016/j.jaclp.2024.04.004 38636900

[24] Heeney M Herring A Anderson E . 159 ketamine use for buprenorphine-precipitated opioid withdrawal: A case series of 10 patients. Ann Emergency Med. (2022) 80:S72. doi: 10.1016/j.annemergmed.2022.08.183

[25] Hendy K . Placebo problems: boundary work in the psychedelic science renaissance. In: Labate BC Cavnar C , editors. Plant medicines, healing and psychedelic science: cultural perspectives. Springer International Publishing, Cham (2018). p. 151–66. doi: 10.1007/978-3-319-76720-8_9

[26] Clements JA Nimmo WS Grant IS . Bioavailability, pharmacokinetics, and analgesic activity of ketamine in humans. J Pharm Sci. (1982) 71:539–42. doi: 10.1002/jps.2600710516 7097501

[27] Chong C Schug S Page-Sharp M Ilett K . Bioavailability of ketamine after oral or sublingual administration. Pain Med. (2006) 7:469–9. doi: 10.1111/j.1526-4637.2006.00208_8.x

[28] Rogers AH Zvolensky MJ Ditre JW Buckner JD Asmundson GJG . Association of opioid misuse with anxiety and depression: A systematic review of the literature. Clin Psychol Rev. (2021) 84:101978. doi: 10.1016/j.cpr.2021.101978 33515811

[29] Tormohlen KN Mojtabai R Seiwell A McGinty EE Stuart EA Tobin KE . Co-occurring opioid use and depressive disorders: patient characteristics and co-occurring health conditions. J Dual Diagnosis. (2021) 17(4):296–303. doi: 10.1080/15504263.2021.1979349 PMC10294295 34581663

[30] Peciña M Karp JF Mathew S Todtenkopf MS Ehrich EW Zubieta J-K . Endogenous opioid system dysregulation in depression: implications for new therapeutic approaches. Mol Psychiatry. (2019) 24:576–87. doi: 10.1038/s41380-018-0117-2 PMC6310672 29955162

[31] Connery HS . Medication-assisted treatment of opioid use disorder: review of the evidence and future directions. Harvard Rev Psychiatry. (2015) 23:63. doi: 10.1097/HRP.0000000000000075 25747920

[32] McHugh RK Fitzmaurice GM Votaw VR Geyer RB Ragnini K Greenfield SF . Cognitive behavioral therapy for anxiety and opioid use disorder: Development and pilot testing. J Subst Use Addict Treat. (2024) 160:209296. doi: 10.1016/j.josat.2024.209296 38272120 PMC11060910

[33] Moore BA Fiellin DA Cutter CJ Buono FD Barry DT Fiellin LE . Cognitive behavioral therapy improves treatment outcomes for prescription opioid users in primary-care based buprenorphine treatment. J Subst Abuse Treat. (2016) 71:54–7. doi: 10.1016/j.jsat.2016.08.016 PMC5119533 27776678

[34] Mauro PM Gutkind S Annunziato EM Samples H . Use of medication for opioid use disorder among US adolescents and adults with need for opioid treatment, 2019. JAMA Network Open. (2022) 5:e223821. doi: 10.1001/jamanetworkopen.2022.3821 35319762 PMC8943638

[35] Sassano-Higgins S Baron D Juarez G Esmaili N Gold M . A review of ketamine abuse and diversion. Depress Anxiety. (2016) 33(8):718–27. doi: 10.1002/da.22536 27328618

[36] Ke X Ding Y Xu K He H Wang D Deng X . The profile of cognitive impairments in chronic ketamine users. Psychiatry Res. (2018) 266:124–31. doi: 10.1016/j.psychres.2018.05.050 29864611

[37] Pal R Balt S Erowid E Erowid F Baggott MJ Mendelson J . Ketamine is associated with lower urinary tract signs and symptoms. Drug Alcohol Depend. (2013) 132:189–94. doi: 10.1016/j.drugalcdep.2013.02.005 23474358

[38] Chang M Juruena MF Young AH . Ketamine cystitis following ketamine therapy for treatment-resistant depression – case report. BMC Psychiatry. (2024) 24:9. doi: 10.1186/s12888-023-05468-3 38166893 PMC10763323

[39] Shahzad K Svec A Al-Koussayer O Harris M Fulford S . Analgesic ketamine use leading to cystectomy: A case report. Br J Med Surg Urol. (2012) 5:188–91. doi: 10.1016/j.bjmsu.2011.06.005

[40] Waxman K Shoemaker WC Lippmann M . Cardiovascular effects of anesthetic induction with ketamine. Anesth Analgesia. (1980) 59:355. doi: 10.1213/00000539-198005000-00007 7189381

[41] Andrade C . Ketamine for depression—Knowns, unknowns, possibilities, barriers, and opportunities. JAMA Psychiatry. (2023) 80:1189–90. doi: 10.1001/jamapsychiatry.2023.3982 37878329

[42] SPRAVATO . (esketamine) approved in the U.S. as the first and only monotherapy for adults with treatment-resistant depression (2025). JNJ.com. Available online at: https://www.jnj.com/media-center/press-releases/spravato-esketamine-approved-in-the-u-s-as-the-first-and-only-monotherapy-for-adults-with-treatment-resistant-depression (Accessed March 3, 2025).

